# Autosomal Recessive Keratoderma-Ichthyosis-Deafness (ARKID) Syndrome Is Caused by *VPS33B* Mutations Affecting Rab Protein Interaction and Collagen Modification

**DOI:** 10.1016/j.jid.2016.12.010

**Published:** 2017-04

**Authors:** Robert Gruber, Clare Rogerson, Christian Windpassinger, Blerida Banushi, Anna Straatman-Iwanowska, Joanna Hanley, Federico Forneris, Robert Strohal, Peter Ulz, Debra Crumrine, Gopinathan K. Menon, Stefan Blunder, Matthias Schmuth, Thomas Müller, Holly Smith, Kevin Mills, Peter Kroisel, Andreas R. Janecke, Paul Gissen

**Affiliations:** 1 Department of Dermatology, Medical University of Innsbruck, Innsbruck, Austria; 2 Division of Human Genetics, Medical University of Innsbruck, Innsbruck, Austria; 3 MRC Laboratory for Molecular Cell Biology, University College London, London, UK; 4 Institute of Child Health, University College London, London, UK; 5 Institute of Human Genetics, Medical University of Graz, Graz, Austria; 6 The Armenise-Harvard Laboratory of Structural Biology, Department of Biology and Biotechnology, University of Pavia, Pavia, Italy; 7 Department of Dermatology, Academic Teaching Hospital Feldkirch, Feldkirch, Austria; 8 Department of Dermatology, Veterans Affairs Medical Center, University of California, San Francisco, California, USA; 9 California Academy of Sciences, San Francisco, California, USA; 10 Department of Pediatrics I, Medical University of Innsbruck, Innsbruck, Austria; 11 Inherited Metabolic Diseases Unit, Great Ormond Street Hospital, London, UK

**Keywords:** ARC, arthrogryposis renal dysfunction and cholestasis, ARKID, autosomal recessive keratoderma-ichthyosis-deafness, co-IP, co-immunoprecipitation, CORVET, core vacuole/endosome tethering, HOPS, homotypic fusion and vacuole protein sorting, LB, lamellar body, mIMCD3, murine inner medullary collecting duct 3, PPK, palmoplantar keratoderma, SNP, single nucleotide polymorphism, VWS, Vohwinkel syndrome, wt, wild type

## Abstract

In this paper, we report three patients with severe palmoplantar keratoderma associated with ichthyosis and sensorineural deafness. Biallelic mutations were found in *VPS33B*, encoding VPS33B, a Sec1/Munc18 family protein that interacts with Rab11a and Rab25 proteins and is involved in trafficking of the collagen-modifying enzyme LH3. Two patients were homozygous for the missense variant p.Gly131Glu, whereas one patient was compound heterozygous for p.Gly131Glu and the splice site mutation c.240-1G>C, previously reported in patients with arthrogryposis renal dysfunction and cholestasis syndrome. We demonstrated the pathogenicity of variant p.Gly131Glu by assessing the interactions of the mutant *VPS33B* construct and its ability to traffic LH3. Compared with wild-type VPS33B, the p.Gly131Glu mutant VPS33B had reduced coimmunoprecipitation and colocalization with Rab11a and Rab25 and did not rescue LH3 trafficking. Confirming the cell-based experiments, we found deficient LH3-specific collagen lysine modifications in patients’ urine and skin fibroblasts. Additionally, the epidermal ultrastructure of the p.Gly131Glu patients mirrored defects in tamoxifen-inducible VPS33B-deficient *Vps33b*^*fl/fl*^*-ER*^*T2*^ mice. Both patients and murine models revealed an impaired epidermal structure, ascribed to aberrant secretion of lamellar bodies, which are essential for epidermal barrier formation. Our results demonstrate that p.Gly131Glu mutant VPS33B causes an autosomal recessive keratoderma-ichthyosis-deafness syndrome.

## Introduction

Palmoplantar keratoderma (PPK) denotes hyperkeratosis of palms and soles. The inheritance pattern, phenotype characteristics, and location of the hyperkeratosis as well as the presence of additional extracutaneous features form the basis of classification of different types of PPK (Has and Technau-Hafsi, 2016, Lucker et al., 1994, Schiller et al., 2014). Mutations in more than 20 genes have been associated with both isolated and syndromic forms of hereditary PPK. There are two recognized entities of hereditary PPK, which are associated with sensorineural deafness: Vohwinkel syndrome (VWS; Online Mendelian Inheritance in Man [OMIM]. Johns Hopkins University, Baltimore, MD. MIM Number: 124500. http://www.ncbi.nlm.nih.gov/omim/) and keratitis-ichthyosis-deafness syndrome (OMIM. Johns Hopkins University, Baltimore, MD. MIM Number: 148210. http://www.ncbi.nlm.nih.gov/omim/). Both are generally inherited in an autosomal dominant manner and caused by mutations in *GJB2*, encoding a gap junction protein connexin 26. However, VWS does not include keratitis, and the ichthyosis phenotype is less severe in VWS. The phenotypic spectrum of *GJB2*-associated PPK is broad, ranging from diffuse to mutilating PPK with or without ichthyosis and keratitis (Janecke et al., 2001, Janecke et al., 2005, Maestrini et al., 1999, Richard et al., 2002). Very rarely, VWS is due to a maternally inherited point mutation in the mitochondrial *MT-TS1* gene encoding mitochondrial serine tRNA. The resultant deafness is progressive, postlingual, and involves high frequencies. Penetrance and expressivity of this phenotype are variable, suggesting that further modifying environmental and genetic factors are involved (Sevior et al., 1998). Another severe form of autosomal dominant PPK is due to mutations in *LOR,* encoding loricrin, an epidermal cornified envelope component (Schmuth et al., 2004).

Here, we describe three patients with clinical, molecular, and cellular features of an autosomal recessive PPK, associated with ichthyosis and deafness and caused by mutations in *VPS33B*, from here on referred to as autosomal recessive keratoderma-ichthyosis-deafness (ARKID) syndrome.

Mutations in *VPS33B* were previously reported as causal for the rare autosomal recessive multisystem disorder arthrogryposis renal dysfunction and cholestasis (ARC) syndrome (OMIM. Johns Hopkins University, Baltimore, MD. MIM Number: 208085. http://www.ncbi.nlm.nih.gov/omim/) (Gissen et al., 2004, Smith et al., 2012). Patients characteristically present with congenital arthrogryposis often associated with bilateral dislocation of the hips, flexion contractures of the knee joints and rocker-bottom feet, renal proximal tubule dysfunction, neonatal cholestasis, ichthyosis, and severe failure to thrive (Gissen et al., 2006). Additionally, patients display sensorineural deafness, abnormal platelet α-granule biosynthesis, and variable other symptoms including osteopenia, absent corpus callosum, recurrent infections, and mild dysmorphism (Gissen et al., 2006). Although most patients with ARC syndrome die in infancy (Abu-Sa’da et al., 2005), three reported patients with an attenuated form of ARC syndrome survived into childhood (Bem et al., 2015, Smith et al., 2012).

*VPS33B* encodes the Sec1/Munc18 family protein VPS33B. Sec1/Munc18 family proteins are known to regulate targeting and fusion in vesicular trafficking events through their interactions with soluble N-ethylmaleimide-sensitive-factor attachment receptors (Carr and Rizo, 2010). VPS33B yeast homolog Vps33p is a class c vacuolar protein sorting protein required for protein trafficking to the yeast vacuole as part of class c core vacuole/endosome tethering (CORVET) or homotypic fusion and vacuole protein sorting (HOPS) multiprotein tethering complexes (Balderhaar and Ungermann, 2013, Sato et al., 2000). The mammalian homologs of Vps33p are VPS33A and VPS33B (Gissen et al., 2005). VPS33A has been shown to form part of both the mammalian HOPS (Graham et al., 2013, Wartosch et al., 2015) and CORVET complexes (Perini et al., 2014), whereas VPS33B together with its protein partner VIPAR (VPS33B-interacting protein involved in polarity and apical protein restriction, also known as VPS16B and SPE39) performs an independent function from HOPS and CORVET complexes (Banushi et al., 2016).

Recessive mutations in *VIPAS39* (VPS33B interacting protein, apical-basolateral polarity regulator, spe-39 homolog), encoding VIPAR, cause ARC syndrome in cases without VPS33B defects. VPS33B and VIPAR have been shown to form a stable protein complex that is implicated in apical protein localization, phagosome-lysosome and endosome-lysosome fusion, lysosome-related organelle biogenesis, and integrin internalization (Akbar et al., 2011, Bem et al., 2015, Cullinane et al., 2010, Galmes et al., 2015, Xiang et al., 2015). Recently our group has shown that the complex is required for delivery of the collagen-modifying enzyme LH3, encoded by *PLOD3* (procollagen-lysine, 2-oxoglutarate 5-dioxygenase 3), to intracellular membrane-bound pools of collagen through a vesicular trafficking pathway involving Rab10 and Rab25 activity (Banushi et al., 2016). LH3 is a collagen-modifying enzyme whose activity is required for post-translational hydroxylation, galactosylation, and glucosylation of collagen lysine residues (Salo et al., 2006). Such modifications are essential for formation of intermolecular crosslinks and thus stability of collagen structures (Ruotsalainen et al., 2006, Sipilä et al., 2007).

Here we demonstrate a variant in *VPS33B*, to our knowledge previously unreported, which disrupts interaction with known partners and affects LH3 delivery to collagen leading to ARKID syndrome in both homozygous and compound heterozygous forms.

## Results

### Identification of patients with PPK, ichthyosis, and sensorineural deafness

**Figure 1:**
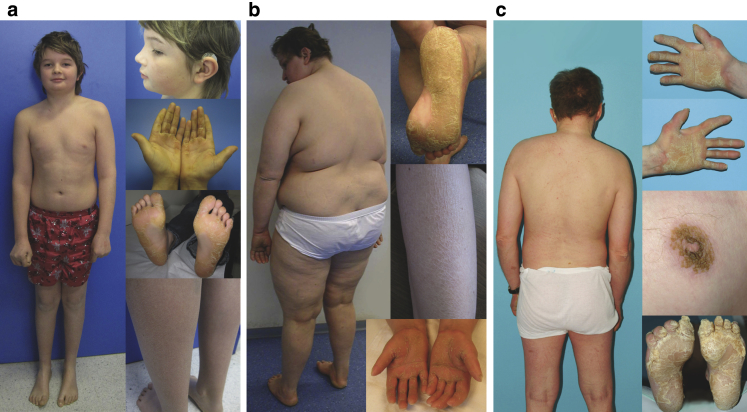
**Clinical features of the three individuals with ARKID syndrome.** (**a**) Patient 1: 13-year-old boy of medium height and build, presenting with diffuse PPK, sensorineural deafness, requiring hearing aids, and generalized fine scaling. (**b**) Patient 2: marked palmoplantar hyperkeratosis, generalized ichthyosis, contractures of fingers, and acromicria in a 43-year-old obese woman with sensorineural hearing loss. (**c**) Patient 3: 49-year-old man of normal weight showing pronounced PPK with the absence of the small toes after autoamputation, generalized fine ichthyosis, perimamillar hyperkeratosis, and sensorineural deafness. ARKID, autosomal recessive keratoderma-ichthyosis-deafness; PPK, palmoplantar keratoderma.

Three patients of Austrian ethnic origin presented with PPK, ichthyosis, and sensorineural deafness (Figure 1). Autosomal recessive inheritance was implied by distant parental consanguinity (third-degree cousins once removed) in one case, and observation of their identical rare surname and highly similar clinical description in the other two patients. Two patients suffered developmental delay and were not able to achieve independent living as adults (patients 2 and 3), whereas one patient (patient 1) had a normal cognitive and motor development.

#### Patient 1

Patient 1 (Figure 1a) is the first child of healthy third-degree cousins once removed; his younger brother is healthy. He was born at term by elective cesarean section weighing 3.35 kg and passed the routine screening for congenital hearing loss. Mild generalized ichthyosis and severe PPK became evident in the first year of life. Moderate sensorineural bilateral symmetrical hearing loss requiring hearing aids was recorded at 9 years old. Hearing loss predominately affected the middle range with hearing thresholds at 50 dB for sensation and 80 dB for word processing at 13 years old. Progressive hearing loss is also indicated by normal newborn screening results but also by gradual worsening of follow-up audiometric testing results. At the age of 13, he had normal anthropometric data (height 167.4 cm, weight 54.4 kg, body mass index 19.4 kg/m^2^) and his psychomotor development was normal. He attended a mainstream school with average results and recently went into training as a carpenter; no IQ tests were performed*.* He had recurrent episodes of epistaxis during his school years (not seen at investigation), and two episodes of macrohematuria at 12 and 13 years old. Minimal relative proteinuria (138 mg protein/g creatinine; reference: 0–100 mg/g) with a total proteinuria of 40 mg/l (reference: 0–150 mg/l) and mildly elevated systolic blood pressure (128/48 mm Hg) were found. He does not receive treatment for this mild elevation in pressure. Elevated alkaline phosphatase bone isoform (>280 IU/l; reference: 30–89 IU/l) was present.

#### Patient 2

Patient 2 (Figure 1b) was born to healthy parents, with no knowledge of consanguinity between them. Two sisters and a brother were healthy. She was born at 41 weeks of gestation weighing 3.73 kg. Congenital bilateral hip subluxation was treated surgically at 6 weeks and 2 years old. PPK and mild-to-moderate generalized ichthyosis presented in infancy. At 8 years old she was diagnosed with bilateral sensorineural hearing loss and received hearing aids; we do not have detailed information regarding this hearing loss. Her psychomotor development was retarded. She was able to walk at 2½ years old. She attended school for the mentally impaired from 7 to 16 years old; no IQ tests were performed*.* Her understanding seems to be normal; her pronunciation is unclear. She is able to perform simple tasks at home, but appears unable to live alone, thought to be due to difficulties in social interactions rather than intellectual disability. Type 2 diabetes mellitus was diagnosed at 32 years old. When investigated at 43 years old, she was obese with height 156 cm, weight 107 kg, body mass index 43.3 kg/m^2^, and head circumference 53 cm, with marked PPK, generalized ichthyosis, contractures of different interdigital joints of all fingers, and acromicria. There was no history of cholestasis, edema, or evidence of bleeding disorders. Laboratory investigations did not reveal signs of kidney and liver disease. Brain magnetic resonance imaging was unremarkable at the age of 35 years. She had a normal female karyotype (550-band resolution), and a normal methylation-sensitive multiplex-ligation-dependent probe amplification analysis result of Prader-Willi syndrome testing.

#### Patient 3

Patient 3 (Figure 1c) was referred at 49 years old for ichthyosis and severe PPK that had already caused mutilation of the small toes by autoamputation. He wore hearing aids for bilateral sensorineural hearing loss diagnosed in adulthood; we do not have detailed information regarding this hearing loss. The patient had clubfeet at birth and trouble walking. He wore an artificial denture, reportedly since childhood. He had a good language understanding by lip reading but hardly intelligible speech. He did not finish school or job training and displayed anxious and shy behavior; no IQ tests were performed. He lived in a special care home. He developed dyspnoea at 52 years old and was diagnosed with interstitial lung fibrosis after approximately 3 months of worsening symptoms; he did not smoke. He died unexpectedly at 59 years old, soon after diagnosis, and no autopsy was conducted. His earlier medical and family history could not be obtained. There was no evidence for cholestasis, edema, or bleeding disorders.

### Identification of mutations in the *VPS33B* gene

As initial screening for VWS candidate genes *GJB2* (NCBI reference sequence: NM_004004.5), *LOR* (NCBI reference sequence: NM_000427.2), and *MT-TS1* (NCBI chromosome MT reference: NC_012920.1) did not reveal pathogenic mutations*,* a whole-genome linkage scan was undertaken. Linkage analysis was based on a subset of 39,000 single-nucleotide polymorphisms (SNPs) with 50 kb distance and a minor allele frequency of ≥0.15. A region of 3 Mb in size on chromosome 15q26 was linked with the disease with a significant logarithm of the odds score of 3.6 under the hypotheses of autosomal recessive inheritance, and a distant relationship of patients 2 and 3, who shared their surname (Supplementary Figure S1 online). The region was flanked by recombinant markers rs12914139 and rs493258 (Supplementary Figure S2 online). Subsequently, two private sequence variants within this linkage region were identified by exome sequencing in patients 2 and 3. Patient 3 had two heterozygous variants in *VPS33B*, an alteration c.[390G>A;392G>A] p.Gly131Glu, not previously identified in patients with ARC and to our knowledge previously unreported, and a splice-site variant c.240-1G>C, previously reported as causing ARC syndrome in homozygous form (Smith et al., 2012). Patient 2 shared the *VPS33B* c.[390G>A;392G>A] variant in homozygous form and also a homozygous *MAN2A2* (NM_006122, c.1400A>G, p.Tyr467Cys) variant. Sanger sequencing confirmed the *VPS33B* c.[390G>A;392G>A] variant in patients 2 and 3. Patient 1 is also homozygous for this variant (Supplementary Figure S3 online). The c.[390G>A;392G>A] variant localized within a 1.8 Mb haplotype that was common to its five disease chromosomes, and which did not contain the *MAN2A2* variant, indicating that the *VPS33B* c.[390G>A;392G>A] variant was derived from a common (Austrian) founder and that patient 3 was most likely compound heterozygous for this variant and the splice-site mutation. Unfortunately, this could not be confirmed, as parental samples were not available for analysis. The two variants were thus segregating appropriately in the pedigrees. Both variants are rare, not listed in the Single Nucleotide Polymorphism Database, Exome Sequencing Project, and Exome Aggregation Consortium databases and absent from 300 anonymous blood donors from Western Austria.

### The p.Gly131Glu variant affects delivery of known VPS33B cargo

Patients with the p.Gly131Glu variant do not present with characteristic ARC features, but share the less well-known features of the disorder, that is, dry, scaly skin and sensorineural deafness. To confirm the p.Gly131Glu variant as causative of the patients’ abnormalities, we investigated its effect, using a previously established cell model of VPS33B deficiency in murine inner medullary collecting duct (mIMCD3) cell lines (Cullinane et al., 2010).

**Figure 2:**
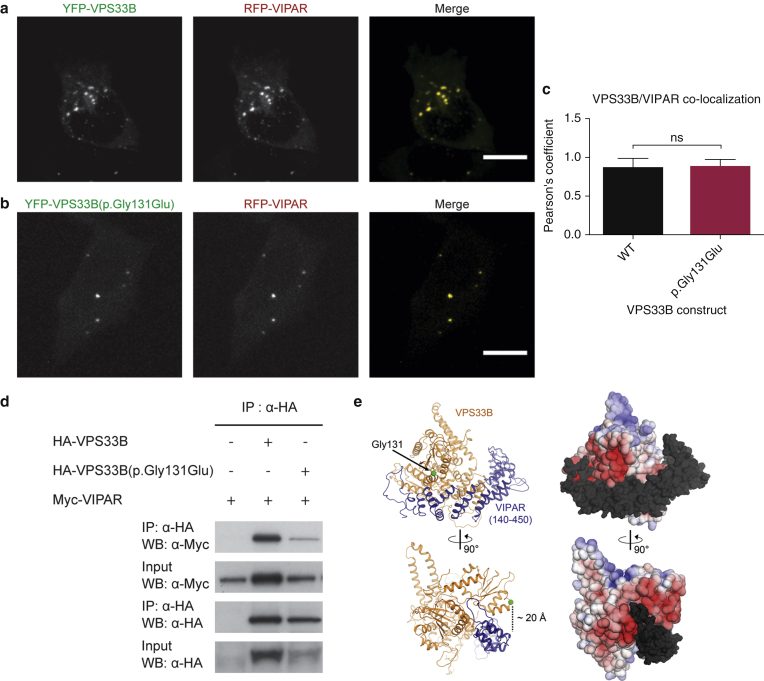
**VPS33B(p.Gly131Glu) does not affect the VPS33B-VIPAR interaction.** (**a, b**) Representative mIMCD3 cells coexpressing RFP-VIPAR and (**a**) wt YFP-VPS33B or (**b**) YFP-VPS33B(p.Gly131Glu). Scale bars = 10 μm. (**c**) Quantification of colocalization between VPS33B constructs and VIPAR. Data are mean ± standard deviation, unpaired *t*-test, ns = nonsignificant. (**d**) Anti-HA co-IP on HEK-293 cell lysates coexpressing HA-VPS33B or HA-VPS33B(p.Gly131Glu) with Myc-VIPAR. (**e**) Mapping of p.Gly131Glu mutation on structural models of the VPS33B-VIPAR complex. Left: VPS33B in orange and alpha solenoid region of VIPAR (residues 140–450) in blue; the disordered N-terminus of VIPAR is omitted. VPS33B residue Gly131 is a green sphere. Right: surface potential of VPS33B, from −5 k_b_Te_c_^−1^ (red) to +5 k_b_Te_c_^−1^ (blue), VIPAR is dark gray. co-IP, co-immunoprecipitation; HA, hemagglutinin; HEK, human embryonic kidney; IP, immunoprecipitation; mIMCD3, murine inner medullary collecting duct 3; RFP, red fluorescent protein; WB, western blot; wt, wild type; YFP, yellow fluorescent protein.

Overexpressed wild-type (wt) VPS33B interacts with VIPAR, whereas ARC-mutant VPS33B has a substantially reduced interaction with VIPAR (Cullinane et al. 2010). In mIMCD3 cells, the p.Gly131Glu variant did not affect colocalization with VIPAR (Figure 2a–c) and co-immunoprecipitation (co-IP) (Figure 2d) indicates that it can still pull down VIPAR, suggesting that the p.Gly131Glu change does not interfere with VPS33B tertiary structure stability or VPS33B-VIPAR interaction. Our results are supported by in silico homology modeling, which demonstrated that residue Gly131 localizes on the VPS33B molecular surface (Figure 2e), in a solvent-exposed region unlikely to be involved in the VPS33B-VIPAR interaction site, estimated to be at a distance of over 20 Å in the homology model.

**Figure 3:**
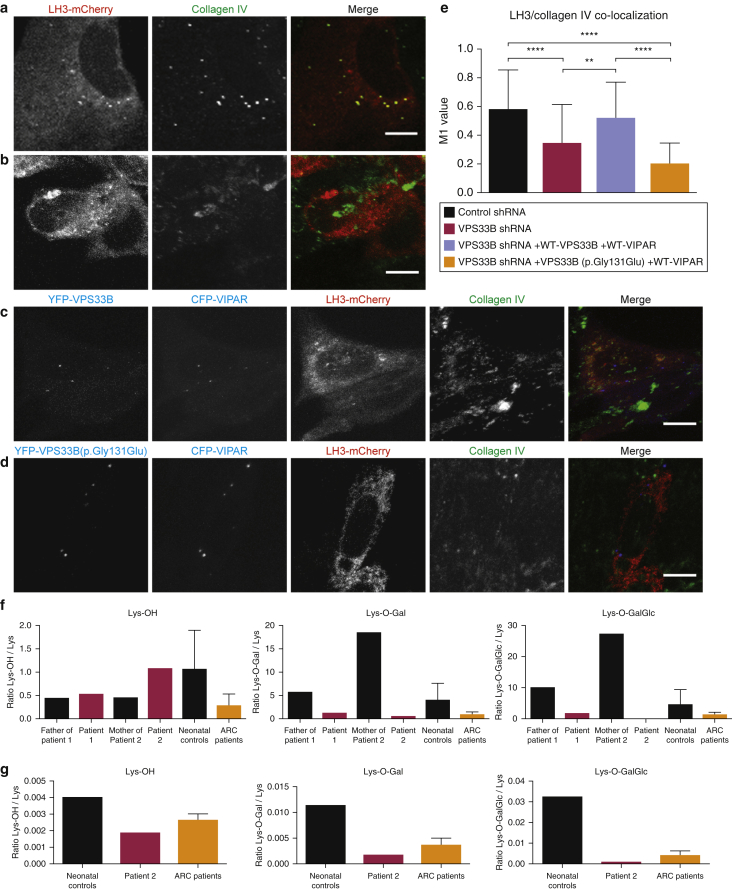
**VPS33B(p.Gly131Glu) cannot rescue defective LH3 delivery in VPS33B-deficient mIMCD3 cells.** (**a–b**) Representative, (**a**) Control, or (**b**) VPS33B shRNA treated mIMCD3 cells expressing LH3-mCherry, stained for collagen IV (**c–d**) Representative VPS33B shRNA-treated mIMCD3 cells expressing (**c**) YFP-VPS33B or (**d**) YFP-VPS33B(p.Gly131Glu) and CFP-VIPAR, LH3-mCherry and stained for collagen IV. (**e**) Quantification of LH3-mCherry/collagen IV colocalization. One-way ANOVA, **P ≤ 0.01, ****P ≤ 0.0001. (**f, g**) LC-MS-MS analysis for relative quantification of hydroxylysines (Lys-OH), galactosyl-hydroxylysines (Lys-O-Gal), and glucosylgalactosyl-hydroxylysines (Lys-O-GalGlc) from (**f**) urine of patient 1 and father, patient 2 and mother, age-matched controls, and patients with ARC, (**g**) collagen I from control, patient 2, and ARC patient skin fibroblasts, statistics not performed as only single patient samples were analyzed. Scale bars = 10 μm. Data are mean ± standard deviation. ANOVA, analysis of variance; ARC, arthrogryposis renal dysfunction and cholestasis; CFP, cyan fluorescent protein; LC-MS-MS, liquid chromatography tandem mass spectrometry; mIMCD3, murine inner medullary collecting duct 3; wt, wild type; YFP, yellow fluorescent protein.

In VPS33B-deficient mIMCD3 cells, unlike wt cells, LH3 is not delivered to intracellular collagen IV carriers and this phenotype can be rescued by cotransfection of wt VPS33B and VIPAR constructs into VPS33B-deficient cells (Banushi et al., 2016). When cotransfected with VIPAR, the VPS33B(p.Gly131Glu) construct could not rescue LH3 colocalization with collagen IV (Figure 3d), unlike the wt construct (Figure 3c and e), suggesting that the p.Gly131Glu variant disrupts the function of VPS33B in vesicular and particularly LH3 trafficking.

Incorrect targeting of LH3 to intracellular collagen causes abnormal post-translational modifications of collagen both in vitro and in vivo (Banushi et al., 2016). In patients with ARC syndrome, defects in LH3-mediated post-translational modifications of collagen lysine residues can be detected by mass spectrometry analysis of urine or cultured fibroblasts. Patients with the p.Gly131Glu variant had reduced levels of hydroxylysines, galactosyl hydroxylysines, and glucosylgalactosyl hydroxylysines in both urine (Figure 3f) and fibroblast lysates (Figure 3g) compared with controls, which is similar to the reduction seen in patients with ARC. In addition, immunostaining of LH3 in the skin of a patient with ARKID showed a decrease in punctate staining in dermal fibroblasts, indicating disrupted LH3 distribution (Supplementary Figure S4a online). These experiments support the conclusion that the p.Gly131Glu variant is pathogenic and causes ARKID syndrome allelic to ARC syndrome.

### The p.Gly131Glu variant affects known VPS33B interactions

**Figure 4:**
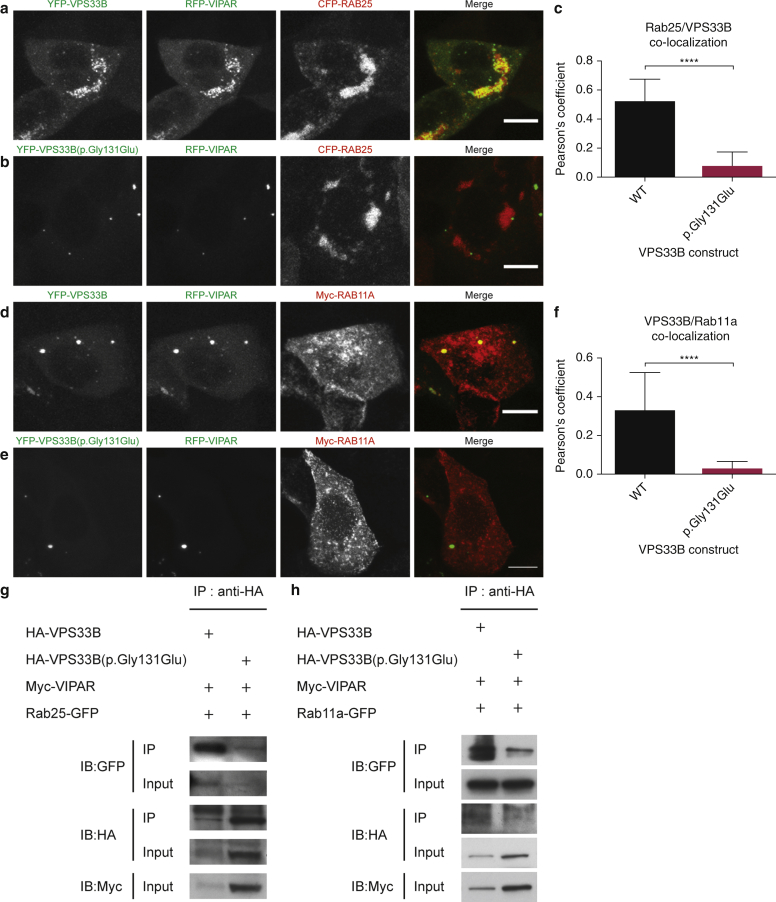
**VPS33B(p.Gly131Glu) alters VPS33B interaction with Rab25 and Rab11a.** (**a, b**) Representative mIMCD3 cells coexpressing wt YFP-VPS33B (**a**) or YFP-VPS33B(p.Gly131Glu) (**b**), RFP-VIPAR and CFP-Rab25. Scale bars = 10 μm. (**c**) Quantification of the colocalization between VPS33B constructs and Rab25 in transfected mIMCD3 cells. Data are mean ± standard deviation, unpaired *t*-test, **** *P* ≤ 0.0001. (**d, e**) Representative mIMCD3 cells coexpressing wt YFP-VPS33B (**d**) or YFP-VPS33B(p.Gly131Glu) (**e**), RFP-VIPAR and Myc-Rab11a, stained with an α-Myc antibody. Scale bars = 10 μm. (**f**) Quantification of the colocalization between VPS33B constructs and Rab11a in transfected mIMCD3 cells. Data are mean ± standard deviation, unpaired *t*-test, **** *P* ≤ 0.0001. (**g**) α-HA co-IP on lysates of HEK293 cells coexpressing HA-VPS33B or HA-VPS33B(p.Gly131Glu) with Myc-VIPAR and Rab25-GFP. (**h**) a-HA co-IP on lysates of HEK293 cells coexpressing HA-VPS33B or HA-VPS33B(p.Gly131Glu) with Myc-VIPAR and Rab11a-GFP. CFP, cyan fluorescent protein; co-IP, co-immunoprecipitation; HA, hemagglutinin; HEK, human embryonic kidney; GFP, green fluorescent protein; IP, immunoprecipitation; mIMCD3, murine inner medullary collecting duct 3; RFP, red fluorescent protein; wt, wild type; YFP, yellow fluorescent protein.

The VPS33B-VIPAR complex cooperates with Rab25, a small GTPase (also known as Rab11c), and the activity of Rab25 is necessary for VPS33B-VIPAR-dependent LH3 delivery to intracellular collagen (Banushi et al., 2016). To determine whether the p.Gly131Glu variant affected LH3 delivery to intracellular collagen through disruption of this interaction, we investigated the colocalization and interaction of VPS33B with Rab25. In mIMCD3 cells, the VPS33B(p.Gly131Glu) construct no longer colocalized with Rab25 (Figure 4a–c) and co-IP experiments showed a lower affinity between VPS33B(p.Gly131Glu) and Rab25 compared with wt (Figure 4g).

VPS33B-VIPAR has also been reported to interact with the Rab11a small GTPase (Cullinane et al., 2010). Although this interaction and the activity of Rab11a do not appear necessary for LH3 delivery to intracellular collagen (Banushi et al., 2016), Rab11a has recently been shown to be essential for skin homeostasis, specifically for epidermal lamellar body (LB) biogenesis (Reynier et al., 2016). LBs are lysosome-related organelles containing, amongst other cargoes, lipids and lipid-modifying enzymes that are secreted by granular layer keratinocytes and are fundamental for the development and maintenance of the epidermal barrier (Feingold, 2012, Feingold and Elias, 2014). In mIMCD3 cells, the VPS33B(p.Gly131Glu) construct no longer colocalized with Rab11a (Figure 4d–f) and co-IP experiments demonstrated a lower affinity between VPS33B(p.Gly131Glu) and Rab11a compared with wt (Figure 4h).

The findings above suggest that the VPS33B region containing the Gly131 residue may be important for interaction of the VPS33B-VIPAR complex with Rab11 family proteins. This region is characterized by numerous negatively charged residues, whose distribution on the VPS33B surface may be critical for molecular interactions (Figure 2e). The introduction of an additional negative charge in this region by the p.Gly131Glu mutation might indeed perturb Rab interactions without inducing changes in the overall VPS33B fold or affecting its interaction with VIPAR, which is predicted to bind in a region distant from Gly131. In ARC syndrome, the VPS33B-VIPAR interaction is disrupted, whereas in patients with ARKID, the VPS33B-VIPAR interaction is intact whilst the Rab interactions are affected. Therefore, it may be that the reduction in interactions with Rab11a and Rab25 is at the core of skin defects in patients with ARC and ARKID syndrome.

### ARKID patient and *Vps33b*^*fl/fl*^*-ER*^*T2*^ mouse skin show impaired epidermal structure

**Figure 5:**
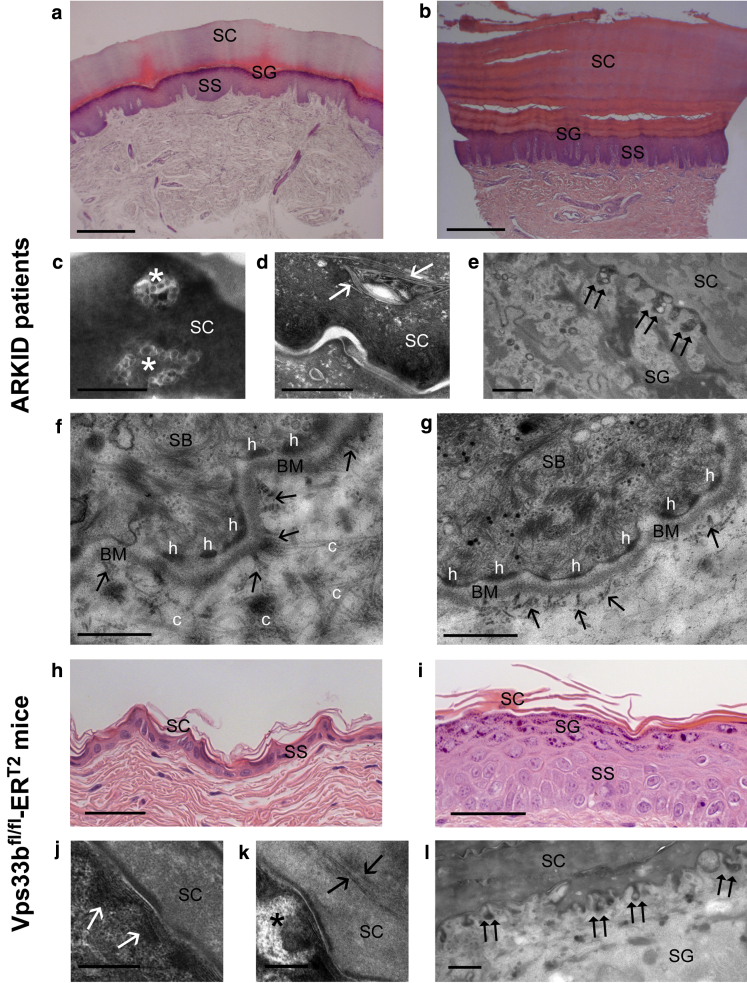
**Similar epidermal defects in ARKID and *Vps33b*^*fl/fl*^*-ER*^*T2*^ mice.** (**a**) Control and (**b**) ARKID palmar skin, H&E, scale bars = 1 mm. (**c–e**) ARKID skin TEM (**c**) LBs (*) and (**d**) lipid bilayers (arrows) within corneocytes, (**e**) inhomogeneous LB secretion (double arrows) and aberrant LBs, scale bars = 0.5 μm. (**f, g**) Dermoepidermal junction in (**f**) control and (**g**) ARKID abdominal skin, anchoring fibrils (arrows), scale bars = 0.5 μm. (**h**) wt and (**i**) *Vps33b*^*fl/fl*^*-ER*^*T2*^ dorsal epidermis, H&E, scale bars = 50 μm. (**j**) wt and (**k**) *Vps33b*^*fl/fl*^*-ER*^*T2*^ epidermis TEM: normal (white arrows) versus entombed bilayers (black arrows), aberrant LBs (*), scale bars = 0.2 μm. (**l**) aberrant LBs at the SG-SC interface (double arrows), scale bar = 2 μm. ARKID, autosomal recessive keratoderma-ichthyosis-deafness; BM, basement membrane; h, hemidesmosomes; H&E, hematoxylin and eosin; LB, lamellar body; SB, stratum basale; SC, stratum corneum; SG, stratum granulosum; SS, stratum spinosum; TEM, transmission electron microscopy; wt, wild type.

Hematoxylin and eosin staining of skin sections of patients with ARKID showed acanthosis with extensive orthohyperkeratosis, hypergranulosis, and elongation of rete ridges without signs of inflammation (Figure 5b). Ultrastructural analysis by transmission electron microscopy revealed LB entombment and inclusion of lipid bilayers within corneocytes (Figure 5c and d), indicating entrapment of nonsecreted LB contents, inhomogeneous LB secretion (Figure 5e) with nonlamellar vesicular contents, and aberrant LB internal structures, suggesting defective loading into the organelles. This is similar to previous findings in ARC syndrome where abnormal LB-like structures within the stratum corneum were attributed to defects in LB secretion (Hershkovitz et al., 2008).

Staining for the LB cargo kallikrein 5 in skin sections of patients with ARKID showed a decrease in kallikrein 5 staining in granular keratinocytes and the stratum corneum (Supplementary Figure S4b), suggesting that VPS33B(p.Gly131Glu) could lead to epidermal barrier defects by impairing LB biogenesis and/or secretion.

Additionally, transmission electron microscopy of the dermoepidermal junction in ARKID abdominal skin sections showed that the basement membrane was deficient (Figure 5g) compared with control sections (Figure 5f). Hemidesmosomes appeared less prominent and the number of anchoring fibrils was clearly reduced. In addition, collagen fibers were less fixed to fibrils and the overall number of dermal collagen fibers appeared reduced, although quantitative analysis was not performed.

We have recently described an inducible *Vps33b* knockout mouse model for ARC syndrome (Bem et al., 2015). Hematoxylin and eosin and transmission electron microscopy analysis of dorsal murine skin biopsies also show acanthosis with hyperkeratosis and hypergranulosis (Figure 5i), as well as abnormal LBs and inhomogeneous secretion (Figure 5k and l). The analogy between ultrastructural defects in patients with ARKID and the ARC murine model indicates similarities in the underlying causative mechanism, strengthening the evidence that the p.Gly131Glu variant causes ARKID abnormalities.

## Discussion

The underlying pathology of the skin phenotype in VPS33B deficiency has previously been attributed to defective LB secretion (Hershkovitz et al., 2008), and Rab11a has recently been shown to be essential for LB biogenesis (Reynier et al., 2016). We demonstrate here that the p.Gly131Glu variant disrupts the interaction of VPS33B-VIPAR with Rab11a and VPS33B deficiency causes abnormal formation of LB structures in mouse and human epidermis, suggesting that this interaction with Rab11a is important for LB biogenesis. Indeed, it may be that defects in intracellular trafficking of cargoes to LB, or defects in LB secretion caused by loss of this interaction are directly affecting keratinocyte differentiation and epidermal morphology in patients with ARKID.

Collagens are a core component of various structures in metazoans such as bones and basement membranes, and are crucial for tissue structure and function, and their assembly requires controlled processing and post-translational modification (reviewed in Ricard-Blum, 2011). LH3-dependent post-translational collagen modifications are crucial for assembly, secretion, and fibril stability of various collagens and homeostasis of the basement membrane (Ruotsalainen et al., 2006, Sipilä et al., 2007, Sricholpech et al., 2012). The homeostasis of the basement membrane composition is essential for the development and maintenance of tissue structure, polarity, and function (Morrissey and Sherwood, 2015, Yurchenco, 2011).

We show that defective LH3 delivery in patients with ARKID is associated with deficiency of anchoring fibrils and less prominent hemidesmosomes indicating defects in the basement membrane. This may explain some of the features observed in these patients with *VPS33B* mutations. This is further supported by overlap in clinical findings, that is, arthrogryposis, sensorineural hearing loss, skin disease, psychomotor delay, and impaired social communication between patients with ARKID and a patient with LH3 deficiency (Salo et al., 2008). We have shown, in VPS33B-deficient mice and mIMCD3 cells, that abnormal LH3 delivery affects collagen homeostasis likely producing alterations in the epidermal basement membrane, changes in which could contribute to the dysregulation of LB biogenesis and/or secretion and function.

VPS33B is ubiquitously expressed, including expression in the vestibulocochlear ganglion (Diez-Roux et al., 2011), and disruption of VPS33B-dependent intracellular protein trafficking and collagen homeostasis may also underlie the sensorineural deafness of patients with ARKID and ARC. Polarized inner ear hair cells are affected by deficiencies in protein trafficking and collagen homeostasis; mutations in the unconventional myosins, for example, myosin VIIA, lead to sensorineural deafness in Usher syndrome (OMIM. Johns Hopkins University, Baltimore, MD. MIM Number: 276900. http://www.ncbi.nlm.nih.gov/omim/), and mutations in collagen IV α5 cause sensorineural deafness in Alport syndrome (OMIM. Johns Hopkins University, Baltimore, MD. MIM Number: 301050. http://www.ncbi.nlm.nih.gov/omim/). Therefore, defects in collagen homeostasis in VPS33B deficiency may explain the similarities of both skin and deafness symptoms in patients with ARKID and ARC.

In summary, the genetic and functional analyses described here show that VPS33B(p.Gly131Glu) is the pathogenic variant that in homozygous, or compound heterozygous form, results in ARKID syndrome by impairing the role of VPS33B in intracellular trafficking.

## Materials and Methods

This study was approved by the institutional review board of the Medical University of Innsbruck and UK National Research Ethics committee (REC13/LO/0168), and complied with the Declaration of Helsinki Principles. Written, informed patient consent was obtained for the experiments. Written consent for the publication of their photographs was obtained from patient 2 and the parents of patient 1; an oral agreement was obtained from patient 3 and so we have not shown his face. Expanded methods and reagent details can be found in the Supplementary Materials and Methods online.

### Linkage analysis and whole exome sequencing

DNA samples were hybridized to HumanCytoSNP-12v2 BeadChip (Illumina, CA) arrays and whole-genome linkage scan was undertaken. Whole-exome sequencing reads were aligned to the human hg19 genome. After removing synonymous SNPs and SNPs with allele frequencies >10% in the 1000 genomes dataset, there were two SNPs left in each sample.

### *Vps33b*^*fl/fl*^*-ER*^*T2*^ mice

*Vps33b*^*fl/fl*^*-ER*^*T2*^ mice have been described (Bem et al., 2015).

### Immunofluorescence and co-IP analysis

mIMCD3 cells from ATCC CRL2123 were cultured as described previously (Banushi et al., 2016). Colocalization, co-IP, and immunoblotting experiments were performed as described previously (Banushi et al., 2016). Paraffin-embedded patient skin sections were incubated in Histo-Clear (National Diagnostics, Atlanta, GA), dehydrated in serial ethanol dilutions, and antigen retrieval performed in citrate retrieval buffer (DAKO, Denmark). Sections were blocked with 3% BSA (Sigma-Aldrich, Irvine, UK), 0.5% tween 20 (Sigma-Aldrich) in phosphate buffered saline; immunofluorescence staining imaging was as described (Banushi et al., 2016).

### In silico homology modeling and mass spectrometry analysis

Modeling, patient urine sample collection, fibroblast culture, and mass spectroscopy were performed as described (Banushi et al., 2016). Samples from patients with ARC (Banushi et al., 2016) were used as positive controls.

### Histology

Five-millimeter punch biopsies were taken from the skin of the palms and abdomen. Specimens were fixed in 4% formaldehyde, embedded in paraffin, sectioned (6 μm), and stained with hematoxylin and eosin. Mouse skin samples were fixed in 10% formalin (Sigma-Aldrich), embedded in paraffin, and sectioned and processed by the Biomedical Research Centre at the Institute of Child Health, London, UK.

### Transmission electron microscopy

Patient samples were analyzed following both reduced osmium tetroxide and ruthenium tetroxide postfixation protocols (Elias, 1996; Gruber et al., 2011). Murine skin biopsies were fixed in 4% glutaraldehyde (TAAB, Berks, UK) in 0.1 M sodium phosphate and incubated with 1% osmium tetroxide (TAAB) 1.5% potassium ferricyanide overnight at 4 °C, then in 1% tannic acid (TAAB) overnight before serial dehydration. Further steps and imaging were as described (Banushi et al., 2016).

## Conflict of Interest

The authors state no conflict of interest.
