# Supplementary material for: Autosomal Recessive Keratoderma-Ichthyosis-Deafness (ARKID) Syndrome Is Caused by VPS33B Mutations Affecting Rab Protein Interaction and Collagen Modification
Source: J Invest Dermatol. 2017 Apr;137(4):845–54. doi: 10.1016/j.jid.2016.12.010 (PMC5358661; doi:10.1016/j.jid.2016.12.010)
Supplement: Supplementary Data [file mmc1.pdf]

SUPPLEMENTAL DATA

Autosomal recessive Keratoderma-Ichthyosis- Deafness (ARKID) syndrome is caused by *VPS33B* mutations affecting Rab protein interaction and collagen modification

Robert Gruber[1,2*], Clare Rogerson[3,4*], Christian Windpassinger[5*], Blerida Banushi[3,4], Anna Straatman-Iwanowska[3,4], Joanna Hanley[3,4], Federico Forneris[6], Robert Strohal[7], Peter Ulz[5], Debra Crumrine[8], Gopinathan K Menon[9], Stefan Blunder[1], Matthias Schmuth[1], Thomas Müller[10], Holly Smith[3], Kevin Mills[4], Peter Kroisel[5+], Andreas R Janecke[2,10+], and Paul Gissen[3,4,11+]

Figure S1.

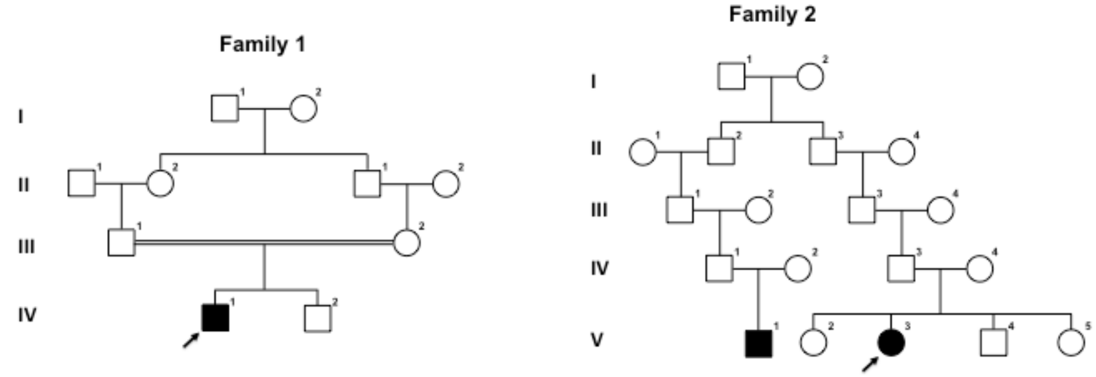

Figure S1. Simplified and hypothetical pedigrees used for linkage analysis. In family 1, the index patient IV-1 (patient 1 in the manuscript), IV-2, III-1 and III-2 were genotyped and included in the linkage analysis. In family 2, the index patient V-3 (patient 2 in the manuscript), her healthy siblings V-2, V-4, V-5, her healthy parents IV-3, IV-4, and patient 3 (V-1) were genotyped and included in the linkage analysis.

Figure S2.

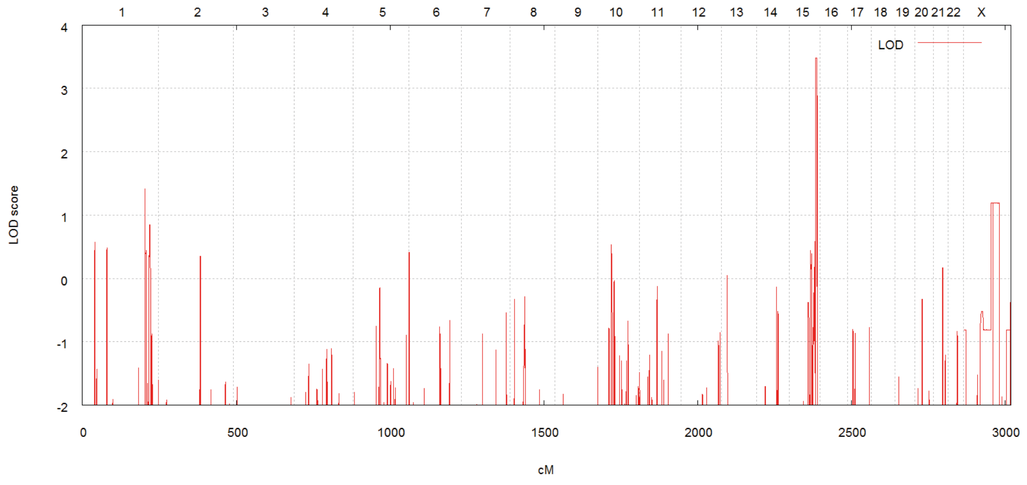

Figure S2. Linkage analysis result based on simplified and hypothetical pedigrees shown in Figure S1. A single, significant region of 3-Mb on chromosome 15q is linked to ARKID. The region is flanked by recombinant markers rs12914139 (NC_000015.10:g.86882728C>A, NC_000015.9:g.87425959C>A, NG_033836.1:g.745718C>A, NM_152336.2:c.3084-105259C>A) and rs4932583 (NC_000015.10:g.91885859A>G, NC_000015.9:g.92429089A>G).

Figure S3.

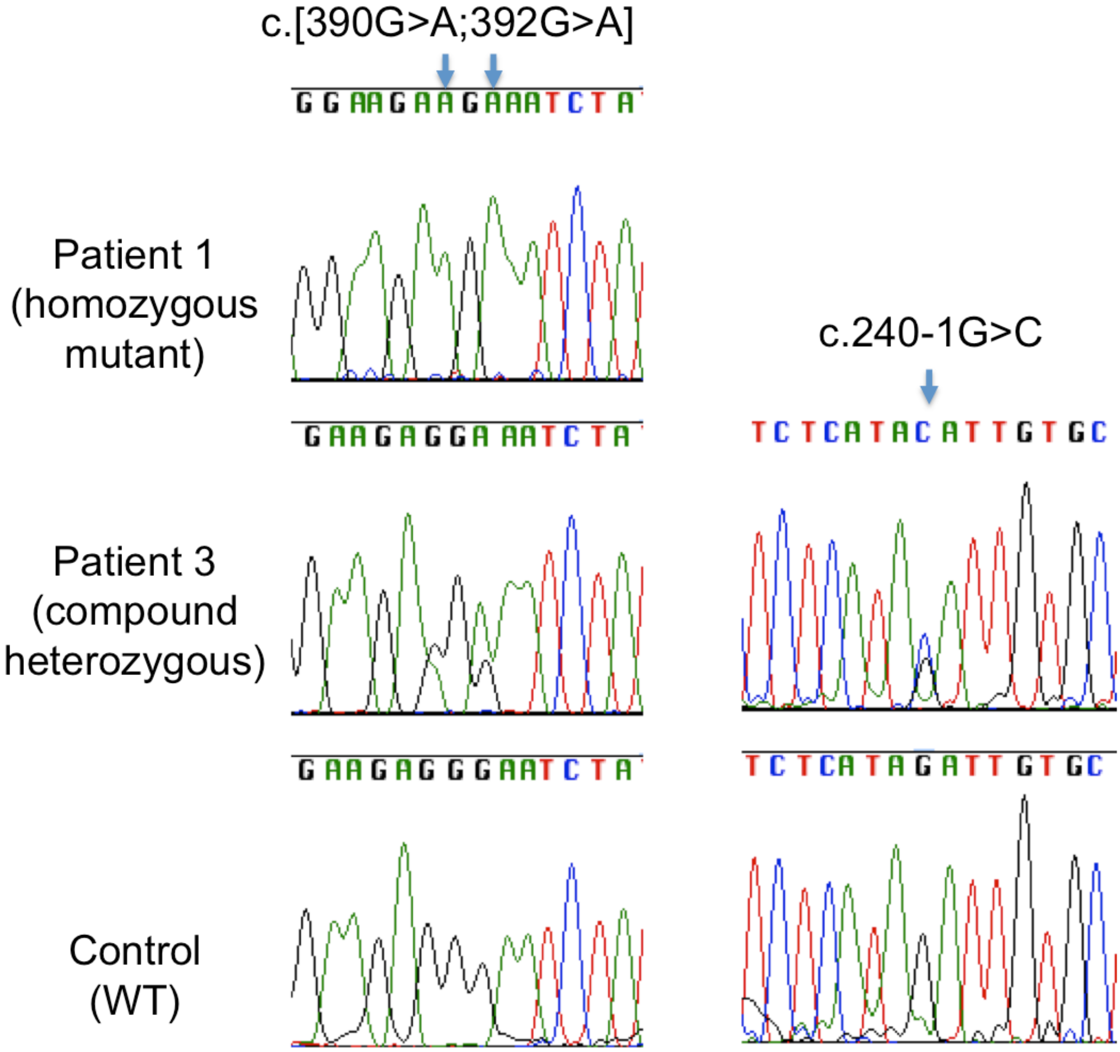

Figure S3. Sanger sequencing of ARKID mutations. The presence of *VPS33B* mutations detected by whole-exome sequencing was confirmed in patients 2 (not shown) and 3, and partial Sanger sequencing of VPS33B in patient 1 detected the same c.[390G>A;392G>A] mutation as present in patient 2.

Figure S4.

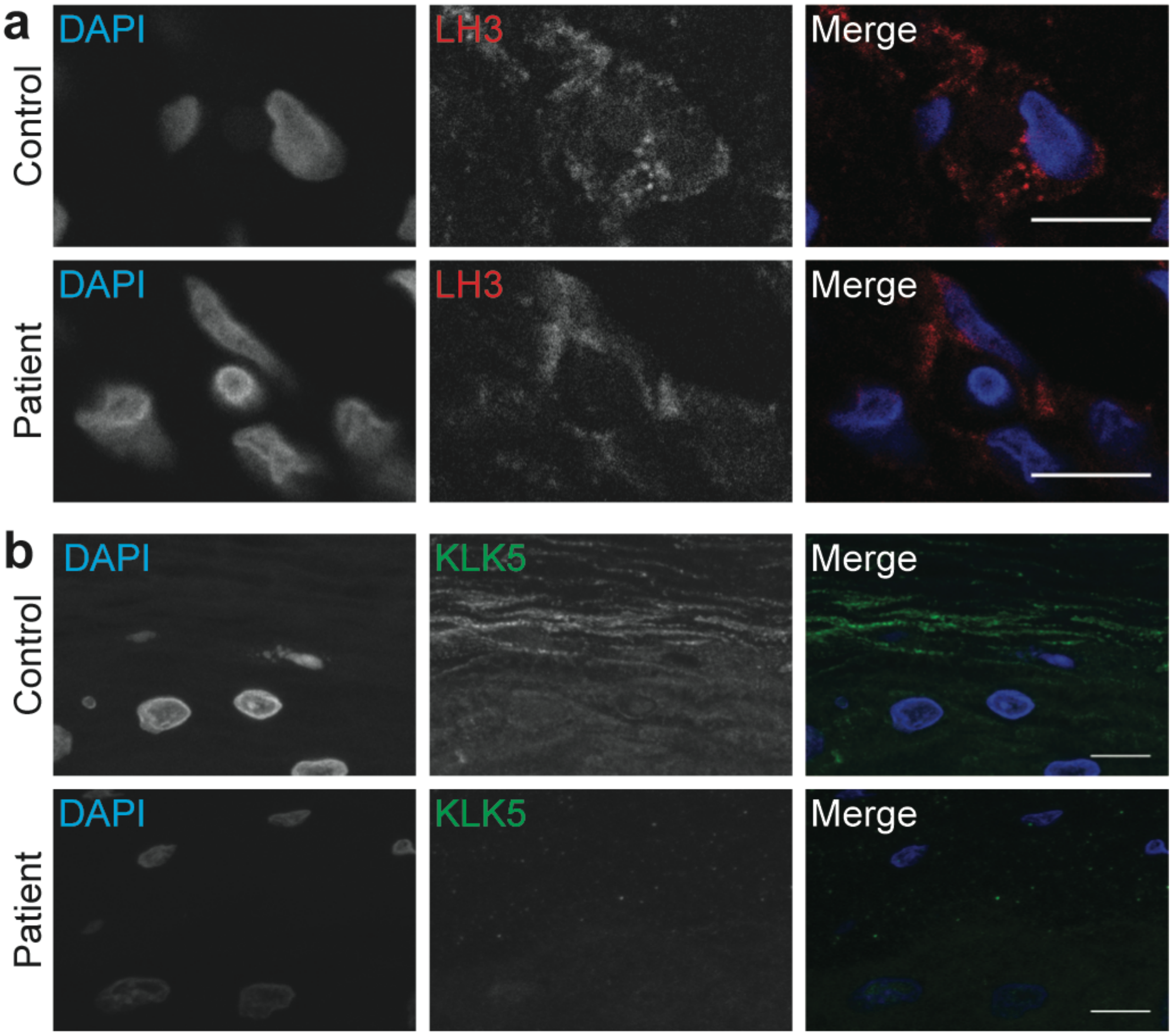

Figure S4. Distribution of cargo proteins in ARKID patient skin sections.

(a) Staining of LH3 in dermal fibroblasts in control and ARKID patient skin sections showing reduced granular distribution of LH3 in ARKID patient skin. (b) Staining of lamellar body cargo protein kallikrein 5 (KLK5) in control and ARKID patient skin sections. The area shown is the junction between the stratum corneum and the stratum granulosum; that patient stratum corneum contains flattened nuclei and shows a reduction in KLK5 staining. Nuclei are counterstained with DAPI. Scale bars = 10 μm.

SUPPLEMENTAL MATERIALS AND METHODS

Linkage analysis and whole exome sequencing and mutation confirmation

The study was approved by the institutional review board of the Medical University of Innsbruck, and complied with the Declaration of Helsinki Principles. DNA samples from 3 affected individuals and 8 family members as shown in Figure S1 were hybridized to HumanCytoSNP-12v2 BeadChip (Illumina, CA) arrays (featuring approximately 300,000 markers). A whole-genome linkage scan was undertaken, and was based on a subset of 39.000 SNPs, selected by an inter-marker distance of 50 kb and a minor allele frequency of ≥0.15, and on autosomal recessive inheritance of the disorder with full penetrance (Figure S2). Whole-exome sequencing of samples from patients 2 and 3 yielded 36.4 million and 20.8 million 100-bp paired-end reads, respectively. Reads were aligned to the human hg19 genome using BWA (version 0.5.9-r16) (Li and Durbin 2009). PCR duplicates were marked using Picard (http://picard.sourceforge.net/index.shtml) and alignments were realigned around indels using GATK (version 1.2-38-g0016c70). Base quality scores were recalibrated and SNPs were identified using the UnifiedGenotyper provided by GATK (McKenna et al. 2010). This resulted in 61,844 and 59,421 variants, respectively, which were annotated using annovar (Wang et al. 2010). Within the linkage region, 56 and 59 high-quality SNPs were found for patients 2 and 3, respectively. After removing synonymous SNPs and SNPs with allele frequencies >10% in the 1000 Genomes dataset, there were 2 SNPs left in each sample. Sanger sequencing used primers 5'-TGACCACAGAGGAACTCCAG-3' (forward) and 5'-TCGTGGTCCTATTCCCATTC-3' (reverse) to confirm the *VPS33B* c.[390G>A;392G>A] variant.

Vps33b$^{fl/fl}$-ER^{T2} mice

Vps33b$^{fl/fl}$-ER^{T2} mice have been described previously (Bem et al. 2015); LoxP sites flank exons 2 and 3 of *Vps33b* and Cre recombinase is under the control of an *ER^{T2}* promoter. Cre

expression was induced by injections of tamoxifen at 6-8 weeks of age and skin biopsies were taken 4-5 weeks later. Controls used were age-matched littermates. All procedures were undertaken with the United Kingdom Home Office approval in accordance with the Animals (Scientific Procedures) Act of 1986.

Antibodies and reagents

The following primary antibodies were used: anti-collagen IV (ab6586, Abcam, UK), anti-GAPDH (ab8245, Abcam, UK), anti-GFP (ab290, Abcam, UK), anti-HA clone HA-7 (H3663, Sigma-Aldrich, UK), anti-KLK5 (MAB7236-SP, R&D Systems, USA), anti-LH3 (11027-1-AP, Proteintech, USA) and anti-c-Myc clone 9E10 (M4439, Sigma-Aldrich, UK). All fluorescent secondary antibodies were Alexa Fluor conjugates (Life Technologies, UK). All plasmids were as previously described (Banushi et al. 2016) except p.Gly131Glu variants which were generated from the previously described fluorescently-tagged and HA-tagged constructs using primers 5’-TGCTTGAGGAAGAAGAAATCTATGGAGATGT-3’ (forward) and 5’-ACATCTCCATAGATTTCTTCTTCCTCAAGCA-3’ (reverse) and the components of the QuikChange XL Site-Directed Mutagenesis Kit (Agilent Technologies) with Platinum Taq HiFi DNA polymerase (Life Technologies).

Immunofluorescence and co-immunoprecipitation analysis

mIMCD3 cells from American Type Culture Collection CRL2123 were cultured as described previously (Banushi et al. 2016) and tested monthly using The MycoAlert™ Mycoplasma Detection Kit (Lonza, UK). Co-localization, co-IP and immunoblotting experiments were performed as described previously (Banushi et al. 2016); constructs were co-transfected into mIMCD3 cells with JetPrime (Polyplus transfection) or HEK293 cells with Lipofectamine2000 (Life Technologies). Representative images are shown in all experiments. Co-localization was measured using the Fiji JACoP plugin. For co-IP

experiments cell lysates were incubated with antibodies conjugated to Dynabeads (Invitrogen).

For immunostaining paraffin embedded patient skin sections were incubated in Histoclear, dehydrated in serial ethanol dilutions and antigen retrieval performed in citrate retrieval buffer (DAKO, Denmark) for two 5 min periods in a microwave at full power. Sections were blocked with 3% BSA in PBS with 0.5% Tween 20 and primary antibodies were also diluted in this blocking buffer. Sections were co-stained with DAPI, mounted with ProLong Gold (Life Technologies) and imaged on the SP5 system as described previously (Banushi et al. 2016).

***In silico* homology modeling**

Modeling was performed as described previously (Banushi et al. 2016) based on the available structural data on the homologous human VPS33A-VPS16 complex (PDB ID 4BX9) and the *C. thermophilum* VPS33-VPS16 complex (PDB ID 4JC8). Structural images were created using PyMOL (Schrödinger 2010) and electrostatic potential calculations performed using APBS (Baker et al. 2001).

Mass spectrometry analysis

Urine samples from patients and fibroblasts from patient 2 were cultured and the lysates subsequently analyzed by mass spectroscopy as described previously (Banushi et al. 2016). Samples from ARC patients (Banushi et al. 2016) were used as positive controls.

Histology

Five-millimeter punch biopsies were taken from the skin of the palms and abdomen. Specimens were fixed in 4% formaldehyde, embedded in paraffin, sectioned (6 μm), and then stained with H&E. Mouse skin samples were fixed in 10% formalin and embedded in

paraffin, sectioned and processed for H&E staining by the Biomedical Research Centre at the Institute of Child Health, London, UK.

Transmission electron microscopy

Patient samples were analyzed following both reduced osmium tetroxide and ruthenium tetroxide postfixation protocols (Elias 1996; Gruber et al. 2011). Murine skin biopsies were fixed in 4% glutaraldehyde in 0.1 M sodium phosphate and incubated with 1% osmium tetroxide 1.5% potassium ferricyanide overnight at 4 °C. A further overnight incubation in 1% tannic acid was performed before serial dehydration and further steps were performed and samples imaged as described previously (Banushi et al. 2016).

ADDITIONAL REFERENCES

Baker, NA, Sept, D, Joseph, S, Holst, MJ, McCammon, JA (2001). Electrostatics of nanosystems: application to microtubules and the ribosome. *Proc Natl Acad Sci U S A* 98: 10037–41.

Elias, PM (1996). Stratum corneum architecture, metabolic activity and interactivity with subjacent cell layers. *Exp Dermatol* 5: 191–201.

Gruber, R, Elias, PM, Crumrine, D, Lin, TK, Brandner, JM, Hachem, JP, *et al.* (2011). Filaggrin genotype in ichthyosis vulgaris predicts abnormalities in epidermal structure and function. *Am J Pathol* 178: 2252–63.

Li, H, Durbin, R (2009). Fast and accurate short read alignment with Burrows-Wheeler transform. *Bioinformatics* 25: 1754–60.

McKenna, A, Hanna, M, Banks, E, Sivachenko, A, Cibulskis, K, Kernytsky, A, *et al.* (2010). The genome analysis toolkit: A MapReduce framework for analyzing next-generation DNA sequencing data. *Genome Res* 20: 1297–303.

Schrödinger, L (2010). *The PyMOL Molecular Graphics System*.

Wang, K, Li, M, Hakonarson, H (2010). ANNOVAR: functional annotation of genetic variants from high-throughput sequencing data. *Nucleic Acids Res* 38: e164.
